# Juvenile Retention Polyp in a Teenager

**DOI:** 10.7759/cureus.16455

**Published:** 2021-07-18

**Authors:** Alexander McClanahan, Pablo Palomo, Ana Burleson, Jolanda Denham, Tamarah Westmoreland

**Affiliations:** 1 Medicine, University of Central Florida College of Medicine, Orlando, USA; 2 Gastroenterology, Nemours Children's Hospital, Orlando, USA; 3 Surgery, University of Central Florida College of Medicine, Orlando, USA; 4 Pediatric Surgery, Nemours Children's Hospital, Orlando, USA

**Keywords:** colonoscopy and polypectomy, pediatric surgery, juvenile retention polyp, colonic polyp, prolapsed rectal mass

## Abstract

The proper management of a prolapsed rectal mass in a child or teenager is challenging. Given that the underlying etiology of a prolapsed rectal mass in this population is not always immediately clear, interdisciplinary assessment is often required. Juvenile polyps, more commonly presenting with bleeding than a prolapsed mass, can mimic the appearance of both hemorrhoids and the rectum itself - making a purely clinical diagnosis difficult. Presented here is a case of a prolapsed colorectal polyp in a teenage boy, who underwent manual reduction of the mass, followed by colonoscopy and endoscopic ligation. Further histological evaluation revealed it to be a juvenile retention polyp. Despite the rarity of polyp prolapse as a presenting symptom, this case underscores the importance of considering colonic polyps as the etiology of a prolapsed anorectal mass in a teenager.

## Introduction

Occurring in about 1% of children, polyps are the leading cause of lower intestinal bleeding in children [1]. Up to 90% of colorectal polyps that arise in children are of the juvenile subtype, and 80%-90% of these exist in either the sigmoid colon or rectum [2]. Given the risk of being associated with cancerous polyposis syndromes (Familial adenomatous polyposis, Gardner’s, Turcot’s, to name a few), colonoscopy and polypectomy have increasingly been used in managing children with recurrent bleeding, pain, or other symptoms associated with colorectal polyps [3,4].

However, despite the difficulty in distinguishing the various subtypes of colorectal polyps grossly and clinically, juvenile retention polyps have low malignant potential. These polyps are classified as hamartomas and histologically contain dilated glands and lamina propria inflammation [3]. A retrospective study of 487 children performed at Beijing Children’s Hospital between 2003 and 2010 showed that rectal bleeding was the most common (94%) clinical manifestation of colorectal polyps in children, while polyp prolapse occurred in only 8.8% of children [5]. Further, ultrasound was found to be positive in 72.3% of patients who eventually underwent colonoscopy, suggesting that ultrasound may be a useful tool in confirming the presence of polyps in children presenting with lower intestinal bleeding, anemia, abdominal pain, or prolapse. In a study of 21 Japanese children with colon polyps from 1984 to 1999, it was reported that all patients reported rectal bleeding, and only three experienced prolapses of the mass through the anus [6].

The differential diagnosis for a prolapsed anorectal mass in children includes ileocecal intussusception, prolapsed rectal polyp, prolapsed rectal duplication cyst, prolapsed rectum, and hemorrhoids [7]. Polyps and hemorrhoids differ from rectal prolapse due to the absence of a visible rectal lumen in the former. Rectal prolapse often contains contributory clinical history and can be elicited by chronic constipation [8]. As hemorrhoids may not be directly visible on an exam, Burge et al. describe a method of unmasking hidden hemorrhoid in children using a 20-French Foley catheter during examination under anesthesia [9].

Given the reported rarity of prolapse as a presenting sign in children with colorectal polyps, the next steps in the management of prolapsed colorectal polyps remain uncertain. Further, the gross appearance of a prolapsed colorectal polyp implicitly broadens the differential to include hemorrhoids, rectal prolapse, and more.

## Case presentation

**Figure 1:**
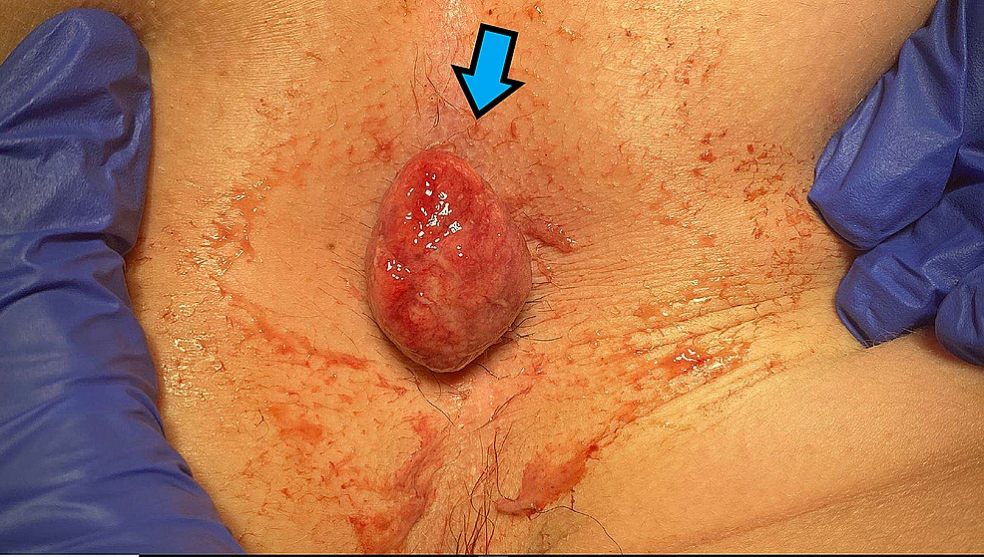
Gross appearance of the juvenile polyp prolapsing through the anus, as present upon initial exam.

A 13-year-old male patient presented to the emergency department (ED) with a prolapsed rectal mass. The patient noticed the mass while attempting a bowel movement, during which he noticed blood on the toilet paper, prompting him and his father to examine the area which contained a round, pink mass protruding from the rectum. A gross image of the mass is shown in Figure 1.

This patient’s history, aside from some constipation as an infant, contained no prior history of rectal prolapse, colorectal polyps, hematochezia, or melena. The patient also denied any abdominal pain, diarrhea, constipation, nausea, or vomiting. Further, the patient’s father was diagnosed with Crohn’s disease in his early 20s. Laboratory studies at three months of age showed reduced hemoglobin A1, indicating a possibility of β-thalassemia trait at the time, which was never confirmed at a later age. On presentation, this patient had a complete blood count (CBC) that showed a mild microcytic anemia. Given that the mass was noted to have a stalk, a rectal polyp was the leading diagnosis prior to any intervention; other diagnoses on the differential included hemorrhoid, rectal prolapse, and inflammatory bowel disease.

Given the patient’s age, noncontributory history, and subtle presentation, determining the appropriate management for this patient presented a challenge in the initial hours of presentation to the ED. However, the polyp was successfully reduced back into the rectum in the ED prior to further intervention. The size of the polyp was likely to pose endoscopic ligation challenges to a gastroenterologist, which subsequently would have warranted recommending surgical resection by a general surgeon.

**Figure 2:**
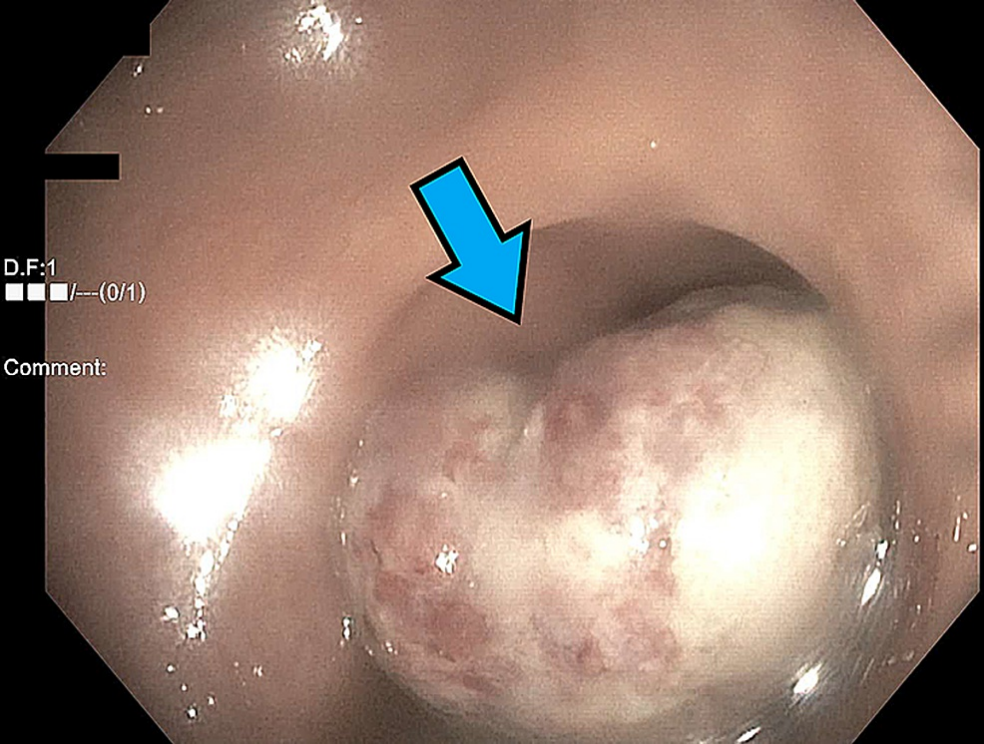
Endoscopic appearance of the juvenile polyp from the sigmoid colon.

**Figure 3:**
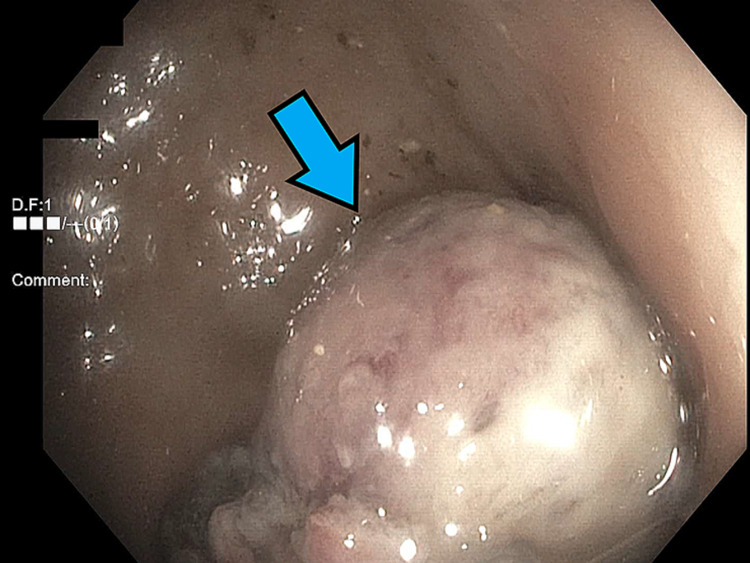
Endoscopic appearance of the juvenile polyp from the descending colon.

Colonoscopy was performed shortly after the mass had been reduced, following bowel prep. Pertinent negatives during the procedure included normal sphincter tone and no palpable rectal lesions. The location of the polyp was found to be in the sigmoid colon, solitary and semi-pedunculated in nature, and measuring 3.3 x 3.0 x 2.8 cm^3^. Endoscopic ligation using a hot snare was performed to remove the polyp. Figures 2, 3 depict the appearance of the polyp in the colon.

Finally, both the gross and histological features of the polyp confirmed the diagnosis as a juvenile retention polyp. Grossly, this polyp was round and pedunculated but lacked the characteristic long pedicles seen in adenomatous polyps. Histologic evaluation of the polyp displayed cystically dilated glands, with relatively equal amounts of glandular and stromal proliferation; this appearance contrasts with adenomatous polyps, which display predominantly glandular proliferation. Lastly, the lack of cellular atypia gave strong evidence that this polyp was indeed benign.

## Discussion

Determining the etiology and appropriate management of a protruding anorectal mass in children can be challenging. As demonstrated by this case, history and physical exam may be insufficient to distinguish between varied conditions such as rectal prolapse, hemorrhoid, and prolapsed colorectal polyp in the child presenting with a mass protruding through the anus. Management for these three conditions differs, underscoring the importance of proper diagnosis.

In this case, the absence of palpable rectal lesions and endoscopic localization of the polyp to the sigmoid colon confirmed the diagnosis of a polyp. Definitive diagnosis and treatment included reduction of the polyp back into the colon, followed by colonoscopy and polypectomy. The colonoscopy allowed for complete colon examination and confirmation of no other polyps or abnormalities.

A spectrum of colorectal polyps exists, ranging from benign to pre-cancerous polyposis syndromes. These polyposis syndromes can be categorized into three broad classes relating to their histology or inheritance: inherited hamartomatous (Peutz-Jeghers, Juvenile polyposis, Cowden’s, Ruvalcaba-Myhre-Smith), inherited adenomatous (Familial adenomatous polyposis, Gardner’s, Turcot’s), and noninherited (Lymphoid polyposis, Cronkhite-Canada) [3]. These syndromes may exhibit extraintestinal manifestations and positive family history, which differentiate them from the simple juvenile polyp. Remes-Troche et al. report that prolapse of polyps in Peutz-Jeghers syndrome is more common in young children (compared to adults) due to less fat in the ischiorectal fossa [10].

Once colonoscopy confirmed that this mass was indeed a prolapsed colorectal polyp, histologically differentiating between the many etiologies of colorectal polyps was equally important, as gross classification is likely insufficient.

## Conclusions

Elucidating the underlying etiology of a prolapsed rectal mass in a child can be challenging and effective treatment depends largely on doing so. Determining the proper course of action relies on interdisciplinary care. Juvenile retention polyps in children commonly present as hematochezia and, less commonly, anal prolapse. Most juvenile retention polyps exist in the rectosigmoid colon. Colonoscopy and polypectomy exist as the standard in removing juvenile retention polyps in children.
